# The cell polarity protein Scrib functions as a tumor suppressor in liver cancer

**DOI:** 10.18632/oncotarget.15713

**Published:** 2017-02-24

**Authors:** Shweta Kapil, Bal Krishan Sharma, Mallikarjun Patil, Sawsan Elattar, Jinling Yuan, Steven X. Hou, Ravindra Kolhe, Ande Satyanarayana

**Affiliations:** ^1^ Department of Biochemistry and Molecular Biology, Molecular Oncology & Biomarkers Program, Georgia Cancer Center, Augusta University, Augusta, GA 30912, USA; ^2^ Stem Cell Regulation and Animal Aging Section, Center for Cancer Research, National Cancer Institute, Frederick, MD 21702, USA; ^3^ Department of Pathology, Augusta University, Augusta, GA 30912, USA

**Keywords:** HCC, nuclear localization, ERK, hippo signaling, Yap1

## Abstract

Scrib is a membrane protein that is involved in the maintenance of apical-basal cell polarity of the epithelial tissues. However, Scrib has also been shown to be mislocalized to the cytoplasm in breast and prostate cancer. Here, for the first time, we report that Scrib not only translocates to the cytoplasm but also to the nucleus in hepatocellular carcinoma (HCC) cells, and in mouse and human liver tumor samples. We demonstrate that Scrib overexpression suppresses the growth of HCC cells in vitro, and Scrib deficiency enhances liver tumor growth in vivo. At the molecular level, we have identified the existence of a positive feed-back loop between Yap1 and c-Myc in HCC cells, which Scrib disrupts by simultaneously regulating the MAPK/ERK and Hippo signaling pathways. Overall, Scrib inhibits liver cancer cell proliferation by suppressing the expression of three oncogenes, Yap1, c-Myc and cyclin D1, thereby functioning as a tumor suppressor in liver cancer.

## INTRODUCTION

*Scrib* (Scrib), along with *Discs large* (Dlg) and *Lethal giant larvae* (Lgl), is an evolutionarily conserved component of a common genetic pathway involved in apical-basal cell polarity [1, 2]. In mammals, cell polarity is established and maintained by at least 3 protein modules (Scrib, Crumbs, and Par). The apical (Crumbs and Par) and basolateral (Scrib) modules function in a mutually antagonistic relationship to regulate various polarization processes such as apical-basal polarity, planar cell polarity, asymmetric cell division and migration [3]. Initial work on these proteins has mainly been focused on identifying their localization in various cell types and their coordination in establishing cell polarity [1, 2]. However, recent studies revealed that by cooperating with diverse partners, these proteins also have independent roles in multiple signaling pathways in a tissue and cell-type specific context [4]. For example, Scrib, by interacting with ZO-2, PHLPP1, Vangl2, APC and ERK [5–9], regulates a number of cellular processes such as cell proliferation, differentiation, apoptosis, stem cell maintenance, migration, and vesicle trafficking [1, 2, 10–13]. Genetic studies indicate that the polarity proteins influence distinct pathways in order to regulate various cellular processes [14].

The tumor-suppressor function of Scrib was first discovered when genetic studies in *Drosophila* revealed that disruption of *Scrib* leads to neoplastic overgrowth of imaginal discs, follicles and brain cells [15, 16]. Scrib localizes to cell–cell junctions and either mislocalization or complete loss of Scrib has a similar phenotype in *Drosophila* [17], indicating that subcellular localization is crucial for proper functioning of Scrib. Concurrent studies have shed some light on a possible role for Scrib in human cancers since human Scrib (hScrib) is targeted for ubiquitin-mediated proteolysis by the E6 oncoprotein from human papillomavirus (HPV) [18], which has a critical role in the development of cervical cancer. In contrast, *Scrib* overexpression suppresses the transforming potential of HPV E6/E7 proteins in rodent epithelial cells [18]. Similar to Scrib, overexpression of *Dlg* in fibroblasts inhibits cell proliferation, suggesting that enhanced expression of cell polarity proteins likely have growth inhibitory effects *in vitro* [19, 20].

In mouse and human studies, down-regulation and cytoplasmic localization of Scrib is commonly observed in colon, ocular, endometrial and breast cancers [21–24]. Mislocalization of not only Scrib, but also Lgl and Dlg, has been associated with cancer progression, suggesting that mislocalization of polarity proteins could have direct implications for cancer development and/or progression [25]. Subsequently, it was demonstrated that Scrib is necessary for prostate homeostasis, and loss of Scrib causes prostate neoplasia due to loss of cell polarity and enhanced activation of Ras/MAPK signaling [26]. A recent study reported that although Scrib is dispensable for normal adult epidermal homeostasis, bi-allelic loss of *Scrib* significantly enhances tumor multiplicity and progression in an autochthonous model of epidermal carcinogenesis, suggesting that Scrib functions as an epidermal tumor suppressor [27].

In contrast to some of the previous reports, a recent study demonstrated that Scrib is overexpressed in the majority of human cancers [28], suggesting that Scrib may not only be down-regulated and mislocalized but also could be overexpressed, and possibly, mislocalized in different cancers. Moreover, contrary to the tumor suppressor function of Scrib in epithelial tissue, loss of Scrib expression delayed the onset of Eμ-myc-driven lymphoma, suggesting a potential oncogenic role of Scrib in Myc-driven lymphoma [29]. Taken together, these studies demonstrate that Scrib not only functions as a tumor suppressor but also as an oncogene, which possibly depends on the context and type of cancer. In light of these contradictory observations in different cancers, we investigated the function of *Scrib* in hepatocellular carcinoma (HCC) cell proliferation, and initiation and progression of liver tumorigenesis. Very surprisingly, we discovered that Scrib not only translocates to the cytoplasm but also to the nucleus in actively proliferating HCC cell lines, and in mouse and human HCC samples. In the current study, we further demonstrated that *Scrib* overexpression (*Scrib OE*) suppressed the proliferation of HCC cells *in vitro*, and *Scrib* deficiency enhanced liver tumor growth *in vivo*.

## RESULTS

### Enhanced expression and nuclear localization of Scrib in human and mouse HCC

**Figure 1:**
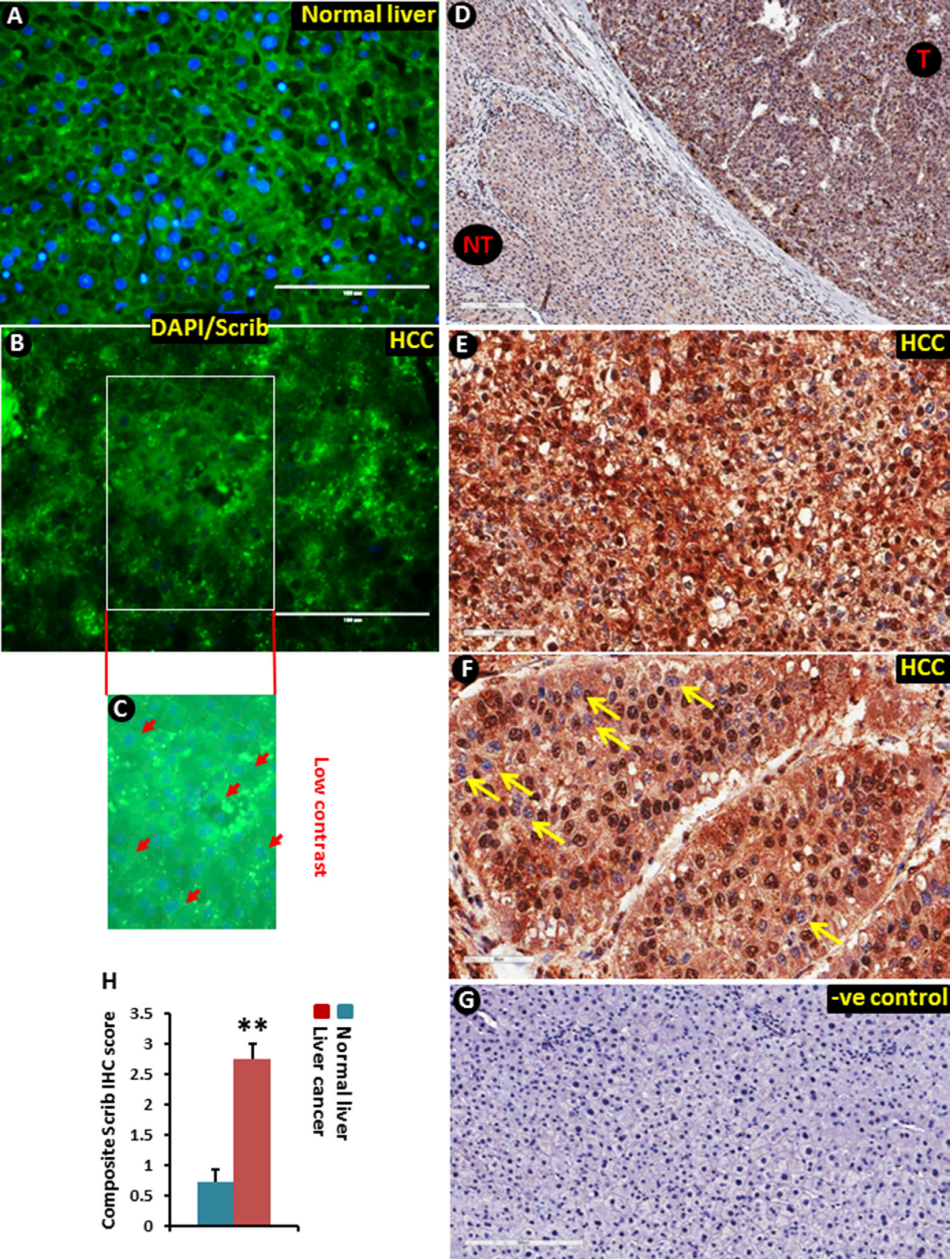
Translocation of Scrib to the nucleus in human liver tumor tissues (**A–B**) Immunofluorescence staining of Scrib protein in normal human liver (A) and HCC (B) samples. (**C**) Low contrast image showing DAPI nuclear staining that was not visible in the merged image (B) due to very intense green (Scrib) fluorescence. Arrow heads point to some of the DAPI stained nuclei. (**D**) Scrib IHC staining in paired human liver tumor and surrounding normal liver tissue. Liver tumor tissues (T) showed stronger Scrib staining pattern compared to non-tumorous (NT) areas; magnification 100x. (**E**) Diffused intense cytoplasmic and nuclear staining of Scrib in liver tumor tissues; magnification 200x. (**F**) Strong intense nuclear staining of Scrib in liver tumor tissues; magnification 200X. All the liver tumor tissues showed strong (3+) diffused cytoplasmic expression of Scrib (*n* = 20/20), and half of the tumors showed strong nuclear expression of Scrib (*n* = 10/20). Arrows indicate nuclei that are not stained for Scrib. (**G**) Negative control for immunohistochemistry. The primary antibody was replaced by normal, non-immune rabbit serum. (**H**) Cytoplasmic and nuclear localization of Scrib was determined by semi-quantitatively assessing the percentage of tumor cells and the staining intensity. Intensity of staining was graded as absent (0), weak (1), moderate (2) and strong (3). The percentage of positive cells was counted in a range of 0-100%. The product of the two scores was calculated as a composite Scrib IHC score. The average composite Scrib IHC score was 2.7525 in the liver cancer group (*n* = 20), and 0.725 in the control normal liver group (*n* = 10). In normal liver tissues ~60% of the cells showed weak (1) cytoplasmic staining of Scrib.

**Figure 2:**
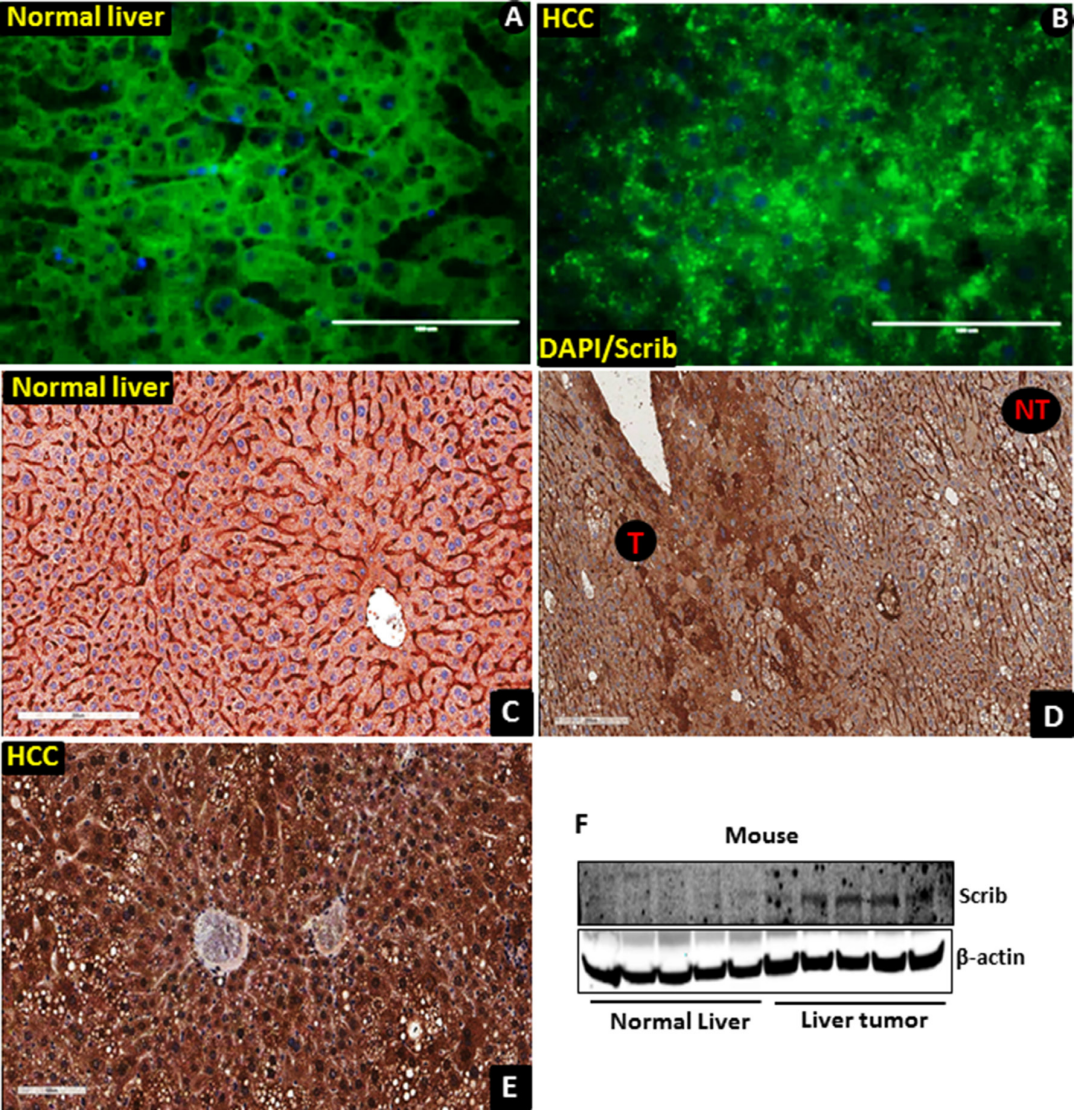
Translocation of Scrib to the nucleus in mouse liver tumor tissues (**A–B**) Staining pattern of Scrib in mouse non-tumorous (A, *n* = 5) and liver tumor (B, *n* = 6) tissues detected by immunofluorescence. (**C**) Strong membranous and weak cytoplasmic expression of Scrib in normal mouse liver tissues (*n* = 5), magnification 200x. (**D**) Scrib IHC staining in paired mouse liver tumor and surrounding normal liver tissue. Liver tumor tissues (T) showed stronger Scrib staining pattern compared to non-tumorous (NT) areas; magnification 100x. (**E**) Diffused intense cytoplasmic and nuclear expression of Scrib in mouse liver tumor tissues, magnification 200X. All the mouse liver tumor tissues showed strong (3+) diffused cytoplasmic and nuclear expression of Scrib (*n* = 6). All the normal liver tissues showed strong membranous (3+) and weak (1) cytoplasmic expression of Scrib (*n* = 5). (**F**) Western blot showing the expression level of Scrib in mouse normal liver and liver tumor tissues.

To determine if Scrib plays any role in liver tumorigenesis, we first analyzed its expression pattern in human HCC samples by immunofluorescence (IF) and immunohistochemical (IHC) staining. The Scrib localization pattern was found to be well structured, and Scrib was predominantly located on the cell membrane in non-tumorous areas of the liver (Figure 1A). In contrast, we detected a highly disorganized and mislocalized pattern for Scrib in human liver tumor samples (Figure 1B–1C), where Scrib was translocated to the cytoplasm and appeared to be also present in the nucleus as well. To further determine whether Scrib expression level and localization was altered between HCC and non-tumorous liver, we performed IHC and detected more intense Scrib staining in the tumorous area compared to the non-tumorous area (Figure 1D). IHC staining also revealed the presence of Scrib in both the cytoplasmic and nuclear compartments of the human liver tumor tissues (Figure 1E–1H). To investigate whether the observations in human liver tumors translate into mouse liver cancer, we induced liver tumors in C57BL/6J wild-type male mice by diethyl nitrosamine (DEN), and performed IF and IHC staining of Scrib. Similar to the human samples, the Scrib expression pattern was well structured, and Scrib was predominantly present on the cell membrane of normal liver, whereas in the liver tumors Scrib was mislocalized and appeared to be present in both the cytoplasm and nucleus (Figure 2A–2B). This was further confirmed by IHC where we clearly detected Scrib localization in both cytoplasm and nucleus, and the staining intensity was stronger in mouse liver tumors compared to normal liver (Figure 2C–2E). Consistent with this observation, we detected increased expression levels of Scrib protein in the liver tumors compared to non-tumorous tissues by Western blotting (Figure 2F). These observations together suggest that Scrib expression was increased in liver tumors, and Scrib not only translocates to the cytoplasm but also to the nucleus when the hepatocytes were transformed.

### Nuclear localization of Scrib in actively proliferating HCC cells

**Figure 3:**
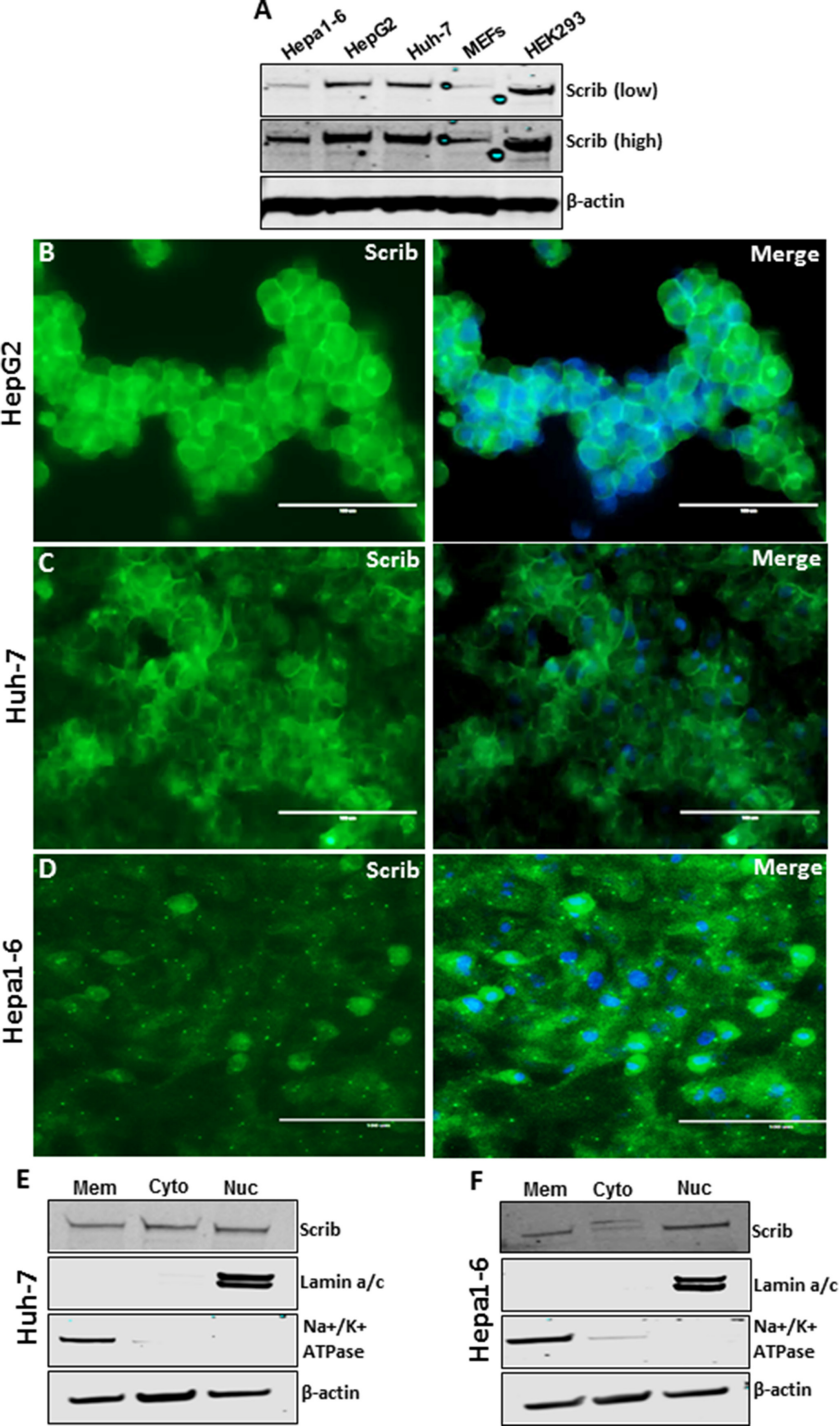
Scrib localization in the nucleus of actively proliferating HCC cells (**A**) Western blot showing the expression level of Scrib and β-actin in the indicated cell lines. (**B–D**) Immunofluorescence staining of Scrib on actively proliferating (~70% confluent) HepG2, Huh-7 and Hepa1-6 cells. (**E–F**) Western blot showing the amount of Scrib present in membrane, cytosolic and nuclear fractions of Huh-7 (E) and Hepa1-6 (F) cells. Lamin a/c and Na^+^/K^+^ATPase were used as nuclear and membrane protein fraction controls.

**Figure 4:**
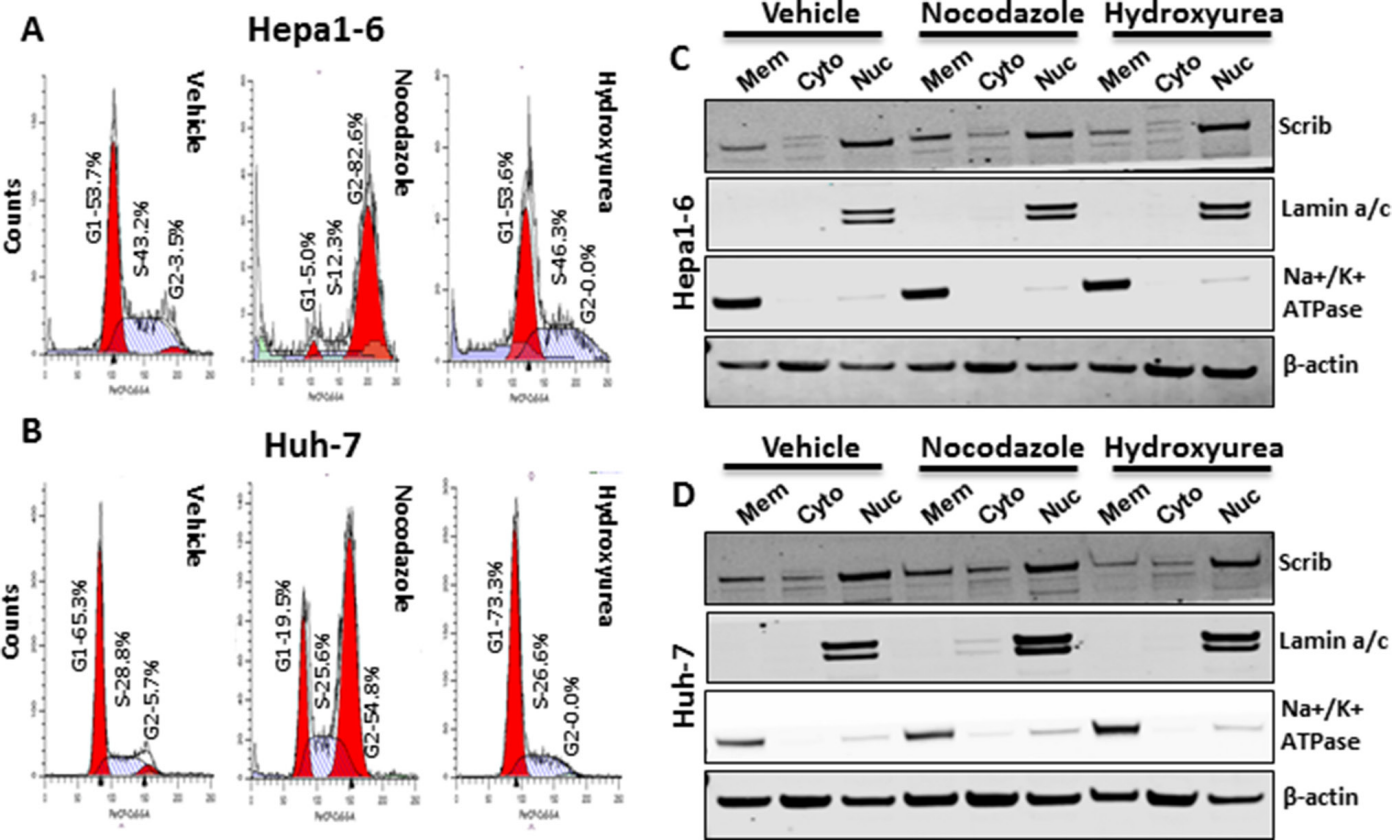
Scrib nuclear localization is independent of cell cycle stage (**A–B**) FACS analysis of Hepa1-6 and Huh-7 cell cycle profiles after treating the cells with Nocodazole or Hydroxyurea or vehicle for 24 h. (**C–D**) Western blots showing the amount of Scrib present in membrane, cytosolic and nuclear fractions of Hepa1-6 (C) and Huh-7 (D) cells after treating the cells with Nocodazole or Hydroxyurea or vehicle for 24 h.

In normal liver, > 90% of the cells are quiescent and Scrib is localized to the cell membrane (Figure 1A, Figure 2A, 2C). In liver tumors, the majority of the cells are in an active proliferative state and Scrib is translocated to the cytoplasm and nucleus (Figure 1E–1F, Figure 2E). Therefore, we reasoned that Scrib translocates to the cytoplasm and nucleus when the cells are in an actively proliferative state. To investigate this possibility, we cultured HCC cell lines and determined Scrib localization by IF and Western blotting. Scrib was strongly expressed in all 3 HCC cell lines, Hepa1-6, HepG2 and Huh-7, and in the kidney epithelial cell line HEK293, but its expression was relatively lower in mouse embryonic fibroblasts (MEFs) compared to other cell lines (Figure 3A). We then stained for Scrib by IF when the cells were ~70% confluent. In Huh-7, HepG2 and Hepa1-6 cells, Scrib was present in both the cytoplasm and nucleus (Figure 3B–3D). To further investigate whether Scrib translocates to the nucleus in actively proliferating cells, we prepared membrane, cytoplasmic and nuclear fractions from Huh-7 and Hepa1-6 HCC cells and detected Scrib in all 3 fractions (Figure 3E–3F), suggesting that Scrib not only translocated to the cytoplasm but also to the nucleus in actively proliferating cells. To further evaluate if Scrib translocation mainly occurs when the cells are at S phase or at G2/M phase of the cell cycle, we arrested cells at G2/M and G1/S phase by nocadazole and hydroxyurea respectively (Figure 4A–4B). We detected higher levels of Scrib in all nuclear fractions compared to the membrane and cytoplasmic fractions (Figure 4C–4D), suggesting that Scrib translocates to the nucleus independent of the specific cell cycle stage.

### Scrib overexpression (Scrib-OE) suppressed HCC cell growth *in vitro* and xenograft tumor growth *in vivo*

**Figure 5:**
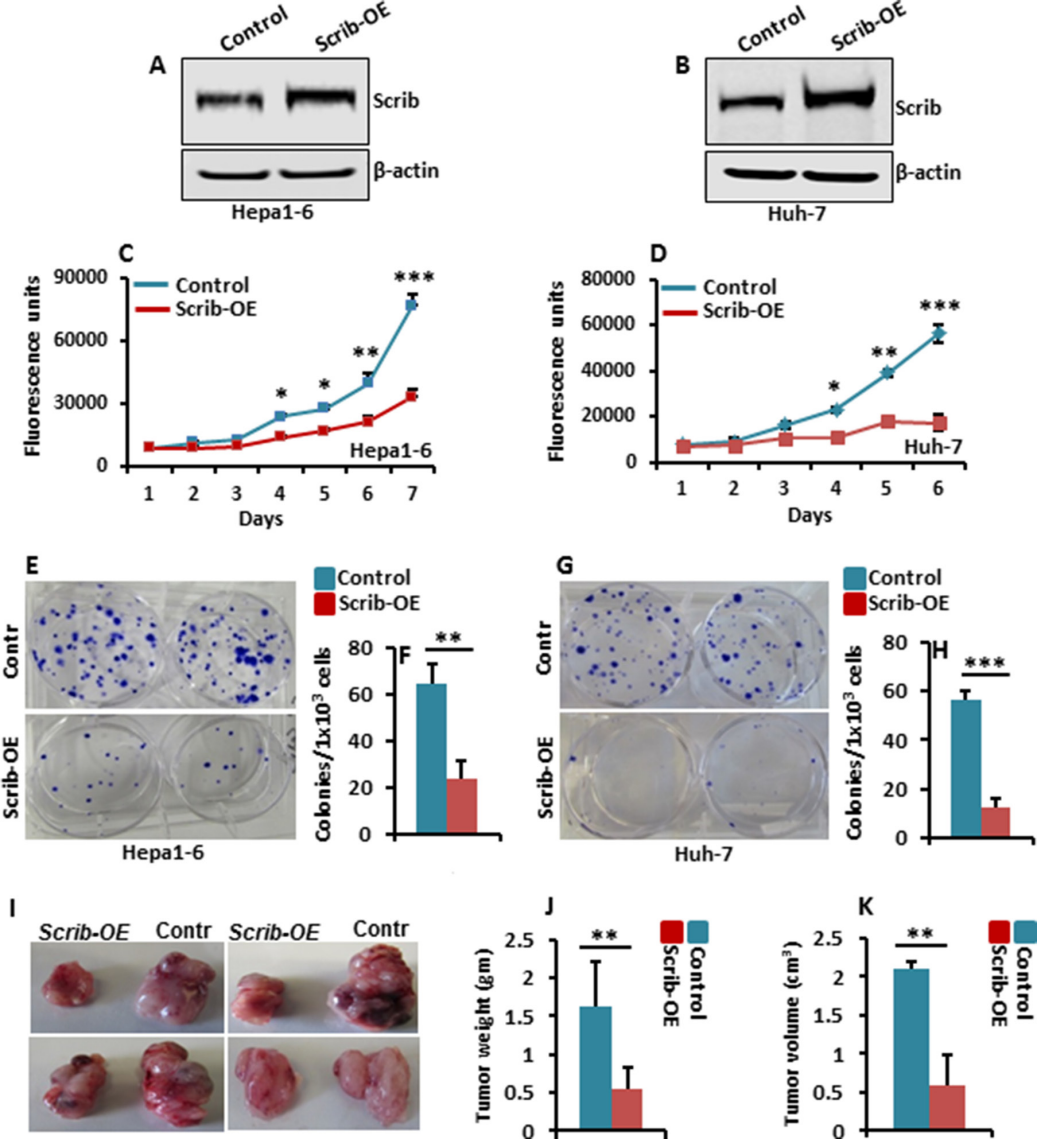
Scrib-OE suppresses HCC cell growth (**A–B**) Western blots of Scrib and β-actin in control and Hepa1-6 and Huh-7 *Scrib-OE* cells. (**C–D**) Proliferation rate of control, Hepa1-6 and Huh-7 *Scrib-OE* cells measured by AlamarBlue proliferation assays. Data are represented as mean ± SD, *p<0.05, **p<0.005, ***p<0.0005. (**E, G**) Representative pictures of crystal violet-stained colonies formed by control and *Scrib-OE* Hepa1-6 (E) and Huh-7 cells (G). (**F, H**) Average number of colonies formed by control and *Scrib-OE* Hepa1-6 (F) and Huh-7 cells (H). Data are represented as mean ± SD, **p < 0.005, ***p < 0.0005. (**I**) Representative photographs showing subcutaneous xenograft growth in nude mice on day 15. Control and *Scrib-OE* Hepa1-6 cells (3 × 10^6^) were injected subcutaneously into 8 week old athymic nude mice. (**J–K**) Bar graphs showing tumor weight (J) and volume of tumors (K) on day 15 (*n* = 5). Data are represented as mean ± SD, **p < 0.005.

Scrib expression was elevated in both mouse and human HCC samples (Figure 1 and 2). To understand the consequence of increased expression of Scrib on HCC cell proliferation, we overexpressed *Scrib* in the HCC cell lines, Hepa1-6 and Huh-7 (Figure 5A–5B), and measured their proliferation and colony formation ability. We detected significantly reduced proliferation (Figure 5C–5D), and impaired colony formation ability (Figure 5E–5H) of *Scrib*-OE Hepa1-6 and Huh-7 cells compared to control cells, suggesting that increased expression of Scrib can have a growth inhibitory effect on HCC cells. To further determine the consequence of *Scrib*-OE on the growth of HCC cells *in vivo*, we performed subcutaneous xenografts in athymic nude mice. Xenograft tumor growth was significantly reduced in *Scrib*-OE cells compared to control cells (Figure 5I–5K), suggesting that Scrib inhibited the growth of HCC cells *in vivo*. These results demonstrate that when Scrib expression is increased, it has a growth inhibitory effect; therefore, Scrib functions as a tumor suppressor in HCC cells.

### DEN-induced liver tumorigenesis is enhanced in liver-specific Scrib-deficient mice

**Figure 6:**
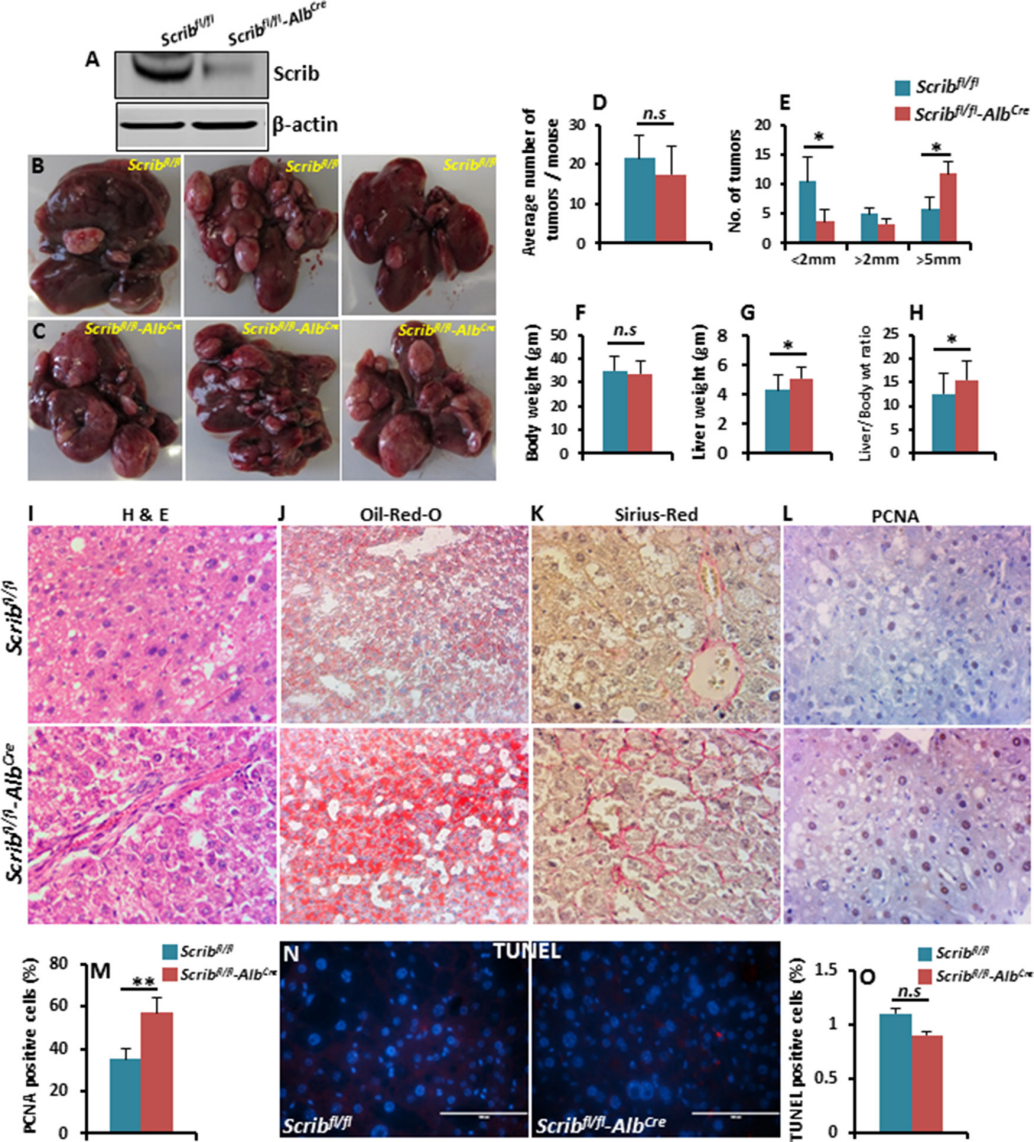
DEN-induced liver tumor growth is accelerated in Scrib-deficient mice (**A**) Western blot showing Scrib levels in 2-month-old *Scrib*^fl/fl^ and *Scrib*^fl/fl^*–Alb*^Cre^ mice confirming *Alb*^Cre^-mediated deletion of *Scrib* in the livers of *Scrib*^fl/fl^*–Alb*^Cre^ mice. (**B–C**) Representative photographs showing liver tumor growth in *Scrib*^fl/fl^ and *Scrib*^fl/fl^*–Alb*^Cre^ mice 40 weeks after DEN treatment. (**D**) Average number of tumors per mouse. (**E**) Liver tumor size in *Scrib*^fl/fl^ (*n* = 9) and *Scrib*^fl/fl^*–Alb*^Cre^ (*n* = 12) mice categorized into 3 groups; small (< 2 mm), medium (2–5 mm) and large (> 5 mm), *p < 0.05. (**F–H**) Body weight (F), liver weight (G) and liver/body weight ratio (**H**) of *Scrib*^fl/fl^ and *Scrib*^fl/fl^*–Alb*^Cre^ mice 40 weeks after DEN treatment, *p < 0.05. Data are represented as mean ± SD. (**I–K**) Representative H&E (I), Oil-Red-O (**J**), and Sirius-Red (K) stained images of *Scrib*^fl/fl^ and *Scrib*^fl/fl^*–Alb*^Cre^ mouse livers 40 weeks after DEN treatment. (**L**) IHC staining of PCNA on the liver tumors of *Scrib*^fl/fl^ and *Scrib*^fl/fl^*–Alb*^Cre^ mice. (**M**) Bar graph showing the percentage of PCNA-positive cells in the liver tumors of *Scrib*^fl/fl^ and *Scrib*^fl/fl^*–Alb*^Cre^ mice, (*n* = 9, *p < 0.05). (**N-O**) TUNEL staining (N) and the percentage of TUNEL-positive cells (O) in the liver tumors of *Scrib*^fl/fl^ and *Scrib*^fl/fl^*–Alb*^Cre^ mice (*n* = 9). Data are represented as mean ± SD.

To further investigate if Scrib functions as a tumor suppressor in a liver cancer model, we crossed *Scrib*^fl/fl^ mice with *Alb*^Cre^ mice to specifically delete *Scrib* from hepatocytes (Figure 6A). We did not detect any spontaneous dysplastic foci, nodules or liver tumors in *Scrib*^fl/fl^*Alb*^Cre^ mice for up to 12 months. Therefore, we induced liver tumorigenesis in *Scrib*^fl/fl^ control and *Scrib*^fl/fl^*Alb*^Cre^mice by the well-established DEN chemical model of liver cancer, [30] and analyzed liver tumor development 40 weeks after DEN treatment. The average number of liver tumors between *Scrib*^fl/fl^ and *Scrib*^fl/fl^*Alb*^Cre^ mice was not significantly changed (Figure 6B–6D). However, further analysis revealed a reduced number of smaller tumors (< 2 mm) and an increased number of larger tumors (> 5 mm) in *Scrib*^fl/fl^*Alb*^Cre^ mice compared to control mice (Figure 6E), suggesting that liver tumor growth was significantly increased in the absence of *Scrib*. Consistent with increased liver tumor growth, liver weight and the liver/body weight ratio were also significantly increased in *Scrib*^fl/fl^*Alb*^Cre^ mice (Figure 6F–6H). Histological analysis of *Scrib*^fl/fl^ and *Scrib*^fl/fl^*Alb*^Cre^ liver tumors revealed large tumor cells with hyperchromatic nuclei in a compact growth pattern (Figure 6I), increased lipid accumulation (Figure 6J) and increased fibrosis (Figure 6K) in *Scrib*^fl/fl^*Alb*^Cre^ liver tumors compared to *Scrib*^fl/fl^ tumors. *Scrib*^fl/fl^*Alb*^Cre^ liver tumors also displayed increased proliferation as revealed by PCNA staining (Figure 6L–6M), but no detectable apoptosis (Figure 6N–6O), indicating that the increased tumor growth in *Scrib*^fl/fl^*Alb*^Cre^ mice was due to increased proliferation rather than changes in apoptosis. These results indicate that *Scrib* deficiency accelerates liver tumor growth, and thus, Scrib functions as a tumor suppressor in liver cancer.

### Scrib-OE impaired ERK activation and down-regulated Yap1, c-Myc and cyclin D1

**Figure 7:**
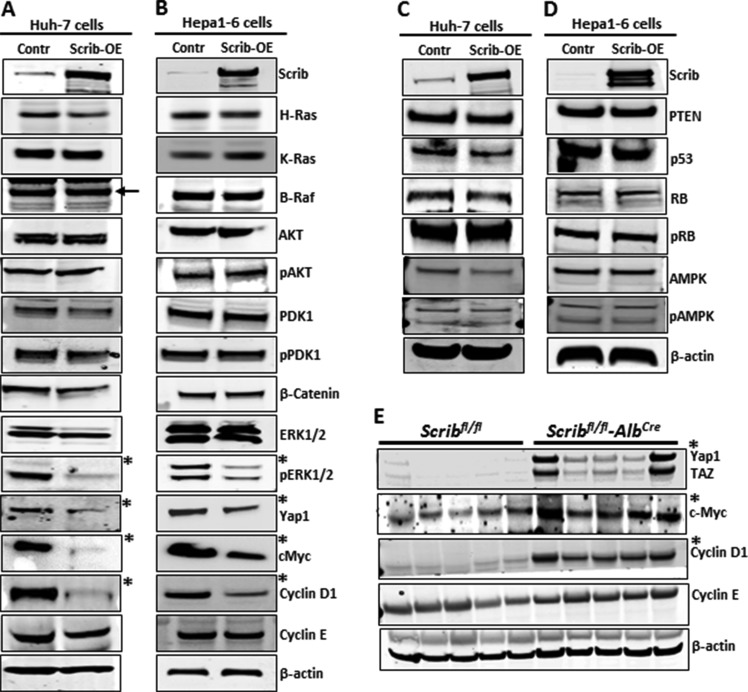
Downregulation of Yap1, c-Myc and cyclin D1 in response to Scrib-OE (**A–D**) Western blots showing the expression levels of indicated proteins in control and *Scrib-OE* Huh-7 (A, C) and Hepa1-6 (B, D) cells. *Indicates detectable difference in expression or activity. (**E**) Western blots displaying the expression level of indicated proteins in the liver tumors of *Scrib*^fl/fl^ and *Scrib*^fl/fl^*–Alb*^Cre^ mice. *Indicates detectable difference in expression.

To understand the molecular mechanism behind Scrib's growth inhibitory function, we analyzed the expression and activity status of some of the oncogenes and tumor suppressors that are commonly deregulated in various cancers including HCC [31–35]. In *Scrib*-OE Huh-7 and Hepa1-6 cells, ERK activation was impaired, and Yap1, c-Myc and cyclin D1 were down-regulated (Figure 7A–7B). However, the expression or activity status of other oncogenes or tumor suppressors that were tested was not significantly altered (Figure 7A–7D). Since the oncogenes Yap1, c-Myc and cyclin D1 were down-regulated in response to *Scrib*-OE we asked how these genes are regulated in the absence of *Scrib*. Analysis of *Scrib*^fl/fl^ and *Scrib*^fl/fl^*Alb*^Cre^ liver tumors revealed enhanced Yap1, c-Myc and cyclin D1 expression in *Scrib*^fl/fl^*-Alb*^Cre^ liver tumors compared to *Scrib*^fl/fl^ liver tumors (Figure 7E). These observations indicate that Scrib inhibits HCC cell proliferation and liver tumor growth by inhibiting the activation of ERK and down-regulating Yap1, c-Myc and cyclin D1.

### Scrib suppressed c-Myc and cyclin D1 expression through ERK

**Figure 8:**
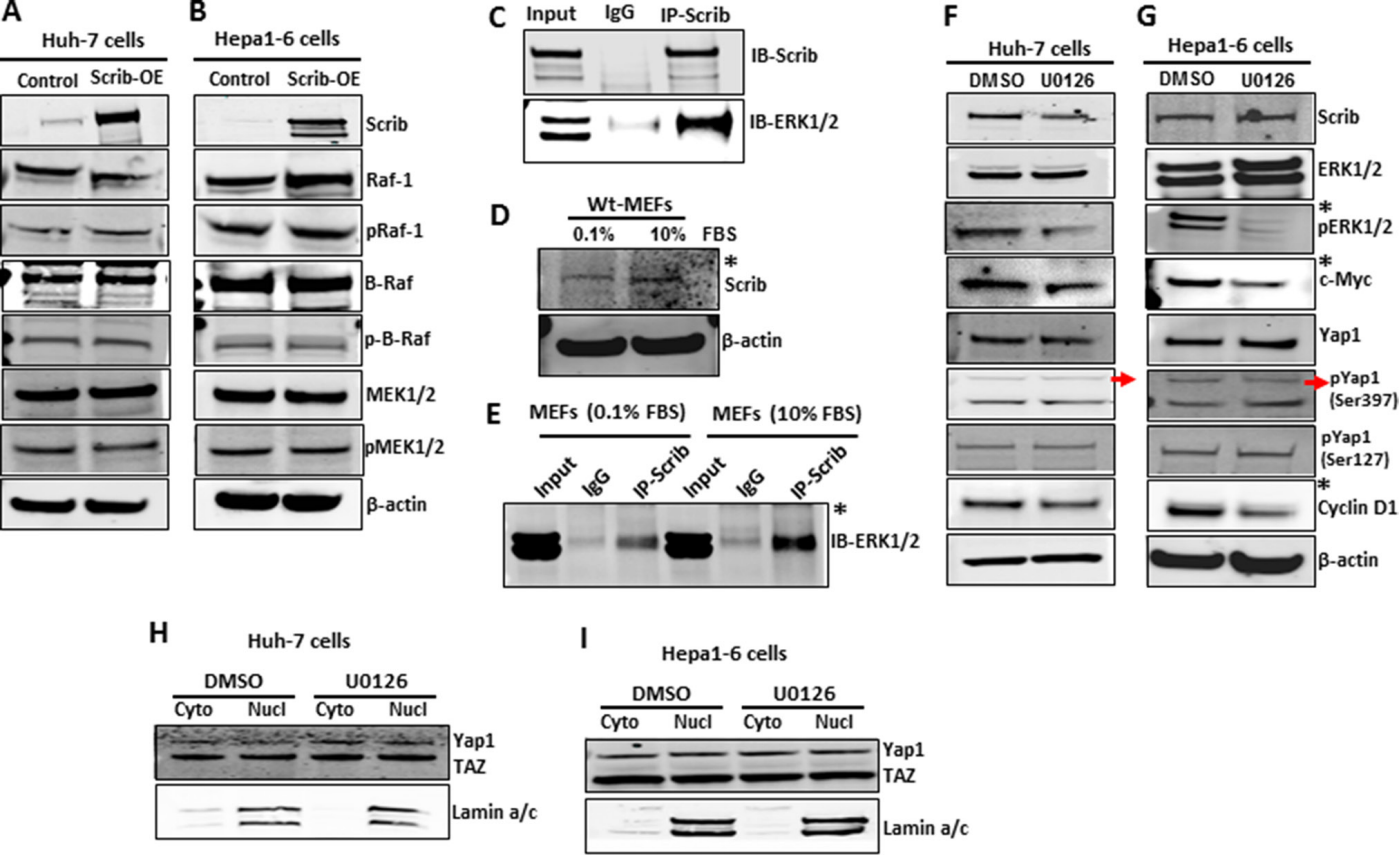
Scrib-mediated downregulation of c-Myc and cyclin D1 is ERK-dependent (**A–B**) Western blots showing the expression and activity status of components of the MAPK/ERK pathway in control and *Scrib-OE* Huh-7 (A) and Hepa1-6 (B) cells. (**C**) Co-IP followed by Western blot showing a direct interaction between Scrib and ERK in Hepa1-6 cells. Input: 10% of IP reaction. (**D**) Expression level of Scrib in serum-starved (0.1% FBS) and serum-stimulated (10% FBS) wild-type MEFs. (**E**) Co-IP followed by Western blot showing increased interaction between Scrib and ERK in serum-stimulated MEFs compared to serum-starved wild-type MEFs. Input: 10% of IP reaction. (**F–G**) Western blots displaying the expression levels of indicated proteins in DMSO or MEK inhibitor U0126-treated Huh-7 (F) and Hepa1-6 (G) cells. *Indicates detectable difference. (**H–I**) Western blots displaying the localization of Yap1 in the cytoplasmic and nuclear fractions of DMSO or MEK inhibitor U0126-treated Huh-7 (H) and Hepa1-6 (I) cells.

We asked if Scrib inhibits ERK phosphorylation by regulating the expression and/or activation of the upstream regulators of ERK in the MAPK pathway [36]. However, we did not detect any significant differences in the expression and activity levels of Raf1, b-Raf and MEK1/2 between control and *Scrib-OE* cells (Figure 8A–8B), suggesting that Scrib regulates ERK activation by alternative mechanisms. Therefore, we further investigated if Scrib directly binds to ERK and inhibits its phosphorylation. We performed Co-IPs and detected a direct interaction between ERK and Scrib in Hepa1-6 cells (Figure 8C). To determine under what condition Scrib interacts with ERK, we conducted Scrib/ERK Co-IPs in serum-starved and serum-stimulated wild-type MEFs. We detected increased expression of Scrib in response to serum stimulation (Figure 8D), and the interaction between Scrib and ERK significantly increased after serum stimulation compared to serum starvation conditions (Figure 8E). We used MEFs instead of HCC cells for serum starvation and stimulation experiments since cancer cells become mitogen independent and do not stay quiescent in response to serum starvation. Our results demonstrate that mitogenic pathways activate ERK, and Scrib controls ERK phosphorylation by directly binding to ERK. Since Scrib suppressed ERK activation, we asked if Scrib down-regulated Yap1, cMyc and cyclin D1 through ERK. Therefore, we treated Hepa1-6 and Huh-7 cells with the MEK inhibitor U0126. We observed reduced ERK activation and down-regulation of c-Myc and cyclin D1 (Figure 8F–8G). However, Yap1 expression, phosphorylation or nuclear localization were not affected in response to ERK inhibition (Figure 8F–8I), suggesting that ERK functions downstream of Scrib and regulates c-Myc and cyclin D1, whereas Scrib-mediated Yap1 suppression is ERK-independent in HCC cells.

### Scrib down-regulated Yap1 independent of ERK and via Hippo pathway

**Figure 9:**
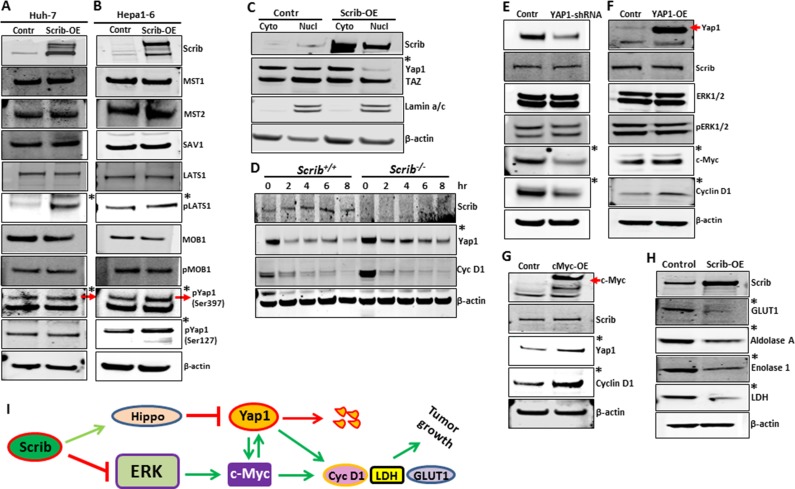
Scrib promotes Yap1 degradation and disrupts c-Myc/Yap1 feed-back loop (**A–B**) Western blots showing the expression and activity status of components of the Hippo signaling pathway in control and *Scrib-OE* Huh-7 (A), and Hepa1-6 (B) cells. (**C**) Western blots displaying the expression of Scrib and Yap1 in the cytoplasmic and nuclear fractions of control and *Scrib-OE* Hepa1-6 cells. (**D**) Cyclohexamide chase experiment followed by Western blotting showing the levels of Scrib, Yap1 and cyclin D1 in *Scrib*^+/+^ and *Scrib*^−/−^ MEFs at the indicated time points. (**E–F**) Western blots displaying the expression levels of indicated proteins in control and Yap1-shRNA (E), and control and *Yap1-OE* Hepa1-6 (F) cells. (**G**) Western blots displaying the expression of Scrib, Yap1 and cyclin D1in control and *c-Myc-OE* Hepa1-6 cells. (**H**) Western blots showing the expression levels of the indicated proteins in control and *Scrib-OE* Hepa1-6 cells. (**I**) Proposed model: Scrib simultaneously regulates the MAPK/ERK and Hippo signaling pathways. Scrib, on one hand, inhibits ERK phosphorylation and activation, and on the other hand, promotes Yap1 degradation via Hippo pathways. In doing so, Scrib breaks the positive feed-back loop between Yap1 and c-Myc, leading to downregulation of c-Myc downstream target genes such as cyclin D1, LDH and Glut1, which promote liver tumor growth.

To investigate if Scrib regulated Yap1 by an ERK-independent pathway, we analyzed the expression and activity levels of the components of the Hippo pathway, which is known to regulate Yap1. The Hippo signaling pathway plays a major role in organ growth control, cell proliferation and tumorigenesis [37]. In the mammalian Hippo pathway, MST/LATS/MOB1/SAV1 constitutes the core protein kinase cascade, that upon activation, phosphorylates and inhibits and/or promotes degradation of Yap1, the major transcriptional effector downstream of the Hippo pathway [38, 39]. A failure to phosphorylate and inhibit Yap1 leads to nuclear translocation and transcriptional induction of the target genes [37]. We asked if Scrib regulated Yap1 through the Hippo pathway and analyzed the expression and activation status of the components of the Hippo pathway and subsequent regulation of Yap1. In response to Scrib-OE, we detected increased phosphorylation and activation of LATS1 (S909), and increased phosphorylation of Yap1, which primes Yap1 for subsequent degradation (Figure 9A–9B). Consistent with this explanation, we detected reduced nuclear Yap1 in *Scrib-OE* cells, whereas the nuclear localization of TAZ, another transcriptional effector of the Hippo pathway, was not affected (Figure 9C), suggesting that Scrib mainly controls Yap1 phosphorylation and degradation in HCC cells. To further investigate if Scrib was indeed required for Yap1 degradation, we evaluated Yap1 protein stability in *Scrib*^+/+^ and *Scrib*^−/−^ MEFs by a cycloheximide chase protein stability assay. We identified increased Yap1 stability in *Scrib*^−/−^ cells compared to *Scrib*^+/+^ cells, whereas cyclin D1 stability was unchanged (Figure 9D). These results together indicate that Scrib controls Yap1 stability by regulating LATS1 phosphorylation.

### Scrib disrupts the positive feed-back loop between c-Myc and Yap1

Our results indicate that Scrib inhibited HCC cell proliferation and liver tumor growth by suppressing Yap1, c-Myc and cyclin D1 via the ERK and Hippo signaling pathways. Since Scrib regulates two separate pathways to suppress Yap1, c-Myc and cyclin D1, we asked whether cross-talk exists between the MAPK/ERK and Hippo/Yap1 pathways, and whether Scrib inhibits both these pathways in order to suppress Yap1, c-Myc and cyclin D1. To investigate the existence of potential cross-talk between the Hippo/Yap1 and ERK/c-Myc pathways, we first knocked down *Yap1* in HCC cells by shRNA, which resulted in down-regulation of c-Myc and cyclin D1 (Figure 9E). Conversely, overexpression of *Yap1* in HCC cells led to increased expression of c-Myc and cyclin D1 (Figure 9F), suggesting that Yap1 regulates c-Myc and cyclin D1 expression in HCC cells. Next, we overexpressed *c-Myc* in HCC cells and detected increased expression of Yap1 and cyclin D1 (Figure 9G), indicating the existence of a positive feed-back loop between Yap1 and c-Myc. To further investigate if c-Myc is the ultimate downstream target of Scrib, we evaluated the expression levels of some of the known c-Myc target genes such as Glut1, Enolase 1 and LDH and detected their down-regulation in *Scrib-OE* cells (Figure 9H). Together, our results highlight the existence of a molecular link (cross-talk) between the MAPK/ERK and Hippo/Yap1 pathways. By simultaneously inhibiting ERK and promoting Yap1 degradation, Scrib appears to disrupt the cross-talk between these two pathways (Figure 9I).

## DISCUSSION

Scrib is a membrane protein containing 16 leucine rich repeat (LRR) domains and four PDZ domains, and it mainly localizes to cell–cell junctions [40]. It has been demonstrated that Scrib is downregulated in various human cancers such as breast, prostate, and colon, and is also mislocalized from cell–cell junctions to the cytoplasm. Moreover, mislocalization of Scrib has been shown to promote breast, lung, colorectal and prostate cancer in various mouse models [21, 22, 26, 41]. Therefore, proper sub-cellular localization of Scrib appears to be essential for its normal cellular function. Very interestingly, we discovered that in HCC cells and in mouse and human liver tumors, Scrib expression is strongly induced and localized to both the cytoplasm and nucleus. Our observation that Scrib can also translocate to the nucleus is very novel, and raises the question whether a membrane protein like Scrib can translocate to the nucleus. Membrane-to-nucleus translocation and the associated nuclear functions have been reported for other proteins [42]. For example, the transmembrane receptor tyrosine kinases ErbB-2 and ErbB-3 of the epidermal growth factor receptor (EGFR) superfamily are known to translocate to the nucleus [42, 43]. In light of these studies, it is not very surprising that a membrane protein such as Scrib can also be localized to the nucleus. Moreover, according to the UniProt/Swiss-Prot the confidence level for the nuclear localization of Scrib is very high (5).

Some previous studies have shown that Scrib is down-regulated in colon, ocular, endometrial and breast cancers [21–24]. However, we found that Scrib expression is strongly induced in mouse and human HCC samples. Our observations are in line with a recent study where it was demonstrated that Scrib is overexpressed in several tumor cell lines, and in the majority of human cancers including HCC [28]. It appears that the expression and localization of Scrib is different from one type of cancer to the other. To understand the consequence of increased expression of Scrib, we overexpressed *Scrib* in HCC cell lines and found that the growth of HCC cells was inhibited, suggesting that when Scrib levels are increased it functions as a tumor suppressor. Not only Scrib, but also elevated levels of some of the cell polarity proteins, result in growth inhibition. For example, overexpression of cell polarity proteins Dlg1 or Dlg3 also inhibits cell proliferation [19, 20]. In various cancers, tumor suppressor genes are often either mutated or down-regulated [44]. If Scrib functions as a tumor suppressor, then, why is its expression strongly induced in HCC? When the cells become cancerous, not only the oncogenes but also some of the growth inhibitors are up-regulated. For example, tumor suppressors such as p16 and p21 are overexpressed in various cancers, and participate in new cellular functions due to increased expression and changes in cellular localization [45, 46].

Previous studies have shown that cytoplasmic translocation of Scrib promotes tumorigenesis [22, 26]. In HCC cells and in mouse and human tumors, we detected Scrib in all three compartments - membrane, cytosolic and nuclear. We discovered that by directly interacting with ERK, and by regulating the Hippo pathway, Scrib suppresses the expression of three oncogenes, Yap1, cMyc and cyclin D1. Liver-specific overexpression of Yap1 or c-Myc or cyclin D1 has been shown to cause spontaneous liver tumorigenesis [33, 34, 47], suggesting that they function as oncogenes in liver tumorigenesis. Although Scrib has been shown to regulate other oncogenes and tumor suppressors such as β-catenin, Akt and PTEN [9, 41, 48], we did not detect any changes in their expression or activity in response to *Scrib*-OE, suggesting that Scrib inhibits liver cancer cell growth by mainly suppressing Yap1 and c-Myc, and consequently their downstream target genes such as cyclin D1, Enolase 1, Aldolase and LDH, which aid cancer cells in proliferation and metabolic reprogramming. Our findings raise another important question; is nuclear translocation of Scrib really essential for its tumor suppressor function? Scrib can directly interact with ERK and inhibit its phosphorylation in the cytosol. Similarly, Scrib can function as a scaffold and bring together the components of the Hippo pathway [49] in the cytosol to increase LATS1 phophorylation, which phosphorylates and promotes Yap1 degradation. Therefore, it appears that the nuclear localization of Scrib may not be essential, at least, for the regulation of the MAPK/ERK and Hippo pathways. Perhaps the cytoplasmic localization of Scrib is sufficient to disrupt the positive feed-back loop between Yap1 and cMyc, and Scrib might participate in additional interactions and regulatory mechanisms in the nucleus. Therefore, future studies need to be directed at understanding the specific oncogenic and tumor suppressor functions of Scrib in the cytosol and nuclear compartments, which will be very challenging.

In contrast to Scrib expression and localization studies in different cancers, which are highly debatable, *Scrib* deficiency has been shown to promote tumorigenesis in a few cancers [22, 26, 50, 51]. We did not detect any spontaneous liver tumorigenesis in *Scrib*- deficient mice, indicating that Scrib is not required for the maintenance of liver structure or function. We found that the average number of liver tumors between *Scrib*^fl/fl^ and *Scrib*^fl/fl^*Alb*^Cre^ mice was not significantly changed but there were an increased number of larger tumors in *Scrib*^fl/fl^*Alb*^Cre^ mice compared to control mice, suggesting that liver tumor growth was increased in the absence of *Scrib*. Therefore, Scrib functions as a tumor suppressor in liver cancer. In contrast to the majority of these studies, a recent study has shown that *Scrib* deficiency delayed Eμ-myc-driven lymphomagenesis [29], indicating that Scrib functions as an oncogene in lymphoma. These studies together demonstrate that the expression, localization and whether Scrib functions as a tumor suppressor or oncogene appears to be rather complex and depends on the cancer type.

## MATERIALS AND METHODS

### Mice

All animal procedures were approved by the Institutional Animal Care and Use Committee at Augusta University, Georgia. *Scrib*^fl/fl^ mice were obtained from Dr. Steven Hou, NCI/NIH. Liver-specific deletion of *Scrib* was achieved by crossing the *Scrib*^fl/fl^ mice with *Alb*^Cre^ mice, which were obtained from Dr. Nahid Mivechi, Augusta University. *Scrib*^fl/fl^ and *Scrib*^fl/fl^-*Alb*^Cre^ mice were genotyped by: GCACACTGGGTATCATGGCTA (forward) and GCAATCTCCAGAGCCTTACAGA (reverse) primers. *Scrib*^+/−^ mice were genotyped by GCACACTGGGTAT CATGGCTA (forward), GCAATCTCCAGAGCCTTAC AGA (WT-reverse) and CCCTTGGAAACCTACAT CCCAA (KO-reverse) primers. To induce liver tumors in *Scrib*^fl/fl^ and *Scrib*^fl/fl^-*Alb*^Cre^ mice, a single dose (25 mg/Kg body weight) of diethylnitrosamine (DEN #N0756; Sigma-Aldrich) was injected intra-peritoneally into 15-day-old pups. The DEN-injected mice were euthanized after 40 weeks, and blood serum, non-tumorous and liver tumor tissues were harvested for further analysis. At the time of collection, we recorded different parameters such as body weight, liver weight, and number of tumors per liver and size of liver tumors.

### Immunohistochemical (IHC) staining of mouse and human HCC samples

To evaluate the expression pattern of Scrib in normal human liver and in liver tumor tissues, we retrieved 20 formalin-fixed, paraffin-embedded cases of liver cancer (American Joint Committee on Cancer stages I–IV) and 10 normal control liver samples from pathology archives of Augusta University after approval from the Institutional Review Board. Seven micrometer thick sections with > 50% tumor tissue from each block were cut and used for Scrib staining. The tissue sections were deparaffinized in xylol and microwave-heated in 0.01M citrate buffer for 16 min. After cooling for 20 min and washing in PBS, endogenous peroxidase was blocked with methanol containing 0.3% hydrogen peroxide for 30 min, followed by incubation with PBS containing 10% normal goat serum for 30 min. For detection of Scrib protein expression, specimens were incubated overnight at 4°C with Scrib primary antibody (#sc-11048, Santa Cruz Biotechnology) at a dilution of 1:50. After 3 washes, sections were subsequently incubated with ready-to-use anti-goat secondary antibody system (DAKO) at room temperature (RT). Peroxidase substrate (3,3′-diaminobenzidine) was used for color development followed by counterstaining with Mayer's haematoxylin solution (# HHS32-1L, Sigma-Aldrich). Tissue sections were finally dehydrated with graded ethanol and mounted by DePex mounting media (# US-15-500, Fisher Scientific). Quantification of Scrib staining on each histological section was performed by two independent pathologists. The same protocol was used for the IHC staining of Scrib in mouse tissues using mouse monoclonal antibody (#sc-55532, Santa Cruz) at a dilution of 1:50.

### Immunofluorescence (IF)

HepG2 and huh-7 cells were cultured on coverslips in 6-well plates at a density of 1 × 10^5^. After 12 h, cells were washed with PBS and fixed in ice-cold acetone:methanol (1:1) for 10 minutes. After 3 washes with cold PBS, cells were blocked with 2% BSA for 1 h at RT and incubated with Scrib (#sc-11048; Santa Cruz) antibody at 4°C overnight. After 3 washes with PBS containing 0.1% Tween 20, coverslips were incubated with Alexa Fluor 488 Donkey anti-goat (#ab150129, Abcam) secondary antibody for 1 h at RT. Cells were washed 3 times with 0.1% PBST, air-dried and mounted with fluorescence mounting medium (#Vectashield H-1200, Vector Laboratories) containing DAPI. The same protocol was used for performing IF on human and mouse tissue sections. A Zeiss Axio fluorescence laser-scanning microscope (Carl Zeiss) was used for capturing the images.

### Cell lines

Hepa1-6 (CRL-1830), Huh-7 (PTA-4583) and HepG2 (HB-8065) cells were purchased from American Type Culture Collection (ATCC). The cells were cultured in DMEM (#SH30022.01, HyClone Laboratories) supplemented with 10% heat-inactivated fetal bovine serum (FBS) (#100106, Gemini Bio-Products) and 1% penicillin/streptomycin solution in a humidified cell culture incubator (#Heracell 150i; Thermo Fisher Scientific) maintained at 37°C, 5% CO_2_, and 20% O_2_. Mouse embryonic fibroblasts (MEFs) were isolated from 13.5-day-old *Scrib*^+/+^ and *Scrib*^−/−^ embryos and cultured in DMEM supplemented with 10% FBS as described previously [52]. Subcellular fractionations from different cell types were prepared using the subcellular protein fractionation kit (#78840, Thermo Scientific) as per the manufacturer's instructions.

### Colony formation and cell proliferation assays

Colony formation and cell proliferation rate of control and *Scrib-OE* cells were performed as described previously [53].

### *In vivo* xenograft assays

Athymic nude mice (strain code #490; Charles River Laboratories) were used for xenograft implantation. Hepa1-6 cells (3 × 10^6^) were implanted subcutaneously in the flank region of each mouse. Control cells were injected in the right flank region, whereas *Scrib-OE* cells were injected into the left flank region of the mice. Mice were monitored daily and euthanized on day 15. Tumor weight and diameter were measured at the time of harvesting. A digital Vernier caliper was used to measure the tumor diameter.

### TUNEL and PCNA staining

Detection of apoptotic cells by TUNEL was performed on formalin-fixed, paraffin-embedded liver sections by ApopTag Peroxidase *In Situ* Apoptosis Detection kit (#S7165; Millipore). PCNA staining was performed on formalin-fixed, paraffin-embedded liver sections by anti-PCNA antibody (1:4000 dilution; ab29; Abcam), DAB and Ultravision Quanto detection system (# TA-060-QHDX; Thermo Fisher). Antigen retrieval was achieved by sub-boiling the sections for 30 min in 0.1 M Citrate Buffer Antigen Retrieval Solution (pH 6.0). Two thousand nuclei were counted and the percentage of PCNA- positive cells was presented. Digital images were acquired by using a Zeiss Axio fluorescence laser-scanning microscope (Carl Zeiss).

### Detection of lipid accumulation and fibrosis in liver tissues

Lipid accumulation and fibrosis were quantified using commercially available kits (Oil Red O stain kit (#IW-3008; Sirus Red stain kit# IW-3012, IHC World) following the manufacturer's instructions. OCT- embedded and paraffin-embedded liver tissue sections were used for Oil-Red-O and Sirus Red staining, respectively. H&E staining was performed as described previously [54]

### Plasmids and vectors

For knockdown and overexpression studies, ContinuumTM Transfection Reagent (Gemini Bio-products # 400-700) was used as per the manufacturer's instructions. pLK45-Scrib (human) was a gift from Ann Hubbard (Addgene Plasmid #37250) [5], pBABE-YAP1 was a gift from Joan Brugge (Addgene Plasmid # 15682) [55], pCDH-puro-cMyc-human was a gift from Jialiang Wang (Addgene Plasmid # 46970) [56], TRMPVneo.Yap.3093 was a gift from Tyler Jacks (Addgene Plasmid #59915) [57], pcDNA3 GFP LIC cloning vector (6D) was a gift from Scott Gradia (Addgene Plasmid #30127), pMSCV PIG (Puro IRES GFP empty vector), and empty backbone was a gift from David Bartel (Addgene Plasmid #21654) [58]. We have generated *Scrib*-dTomato and control dTomato expression vectors using the services of Vector Builder (VB151031).

### Co-immunoprecipitations (Co-IPs)

For Co-IPs, cells were lysed in lysis buffer (50 mM Tris, pH 8.0. 100 mM NaCl, 1 mM EDTA. 1 mMgCl_2_, 10% (v/v) glycerol, 1 % (v/ v) Triton X- 100). Two milligram whole cell lysates were pre-cleared by incubating with 2 μg normal goat IgG (#sc-2028, Santa Cruz) and protein G-agarose beads (#sc-2002, Santa Cruz) at 4°C with gentle shaking. The cell lysate was centrifuged at 2000g for 3 min and the supernatant was collected and transferred into two micro centrifuge tubes and incubated overnight at 4°C with anti-Scrib (#sc-11048 (K-21), Santa Cruz) antibody and normal goat IgG. The immune complex was incubated with protein G-agarose beads for 4 h at 4°C with gentle rotation. After 4 h, beads were pelleted and washed 3 times with cold lysis buffer and reconstituted in 2X protein loading buffer and heated for 5 min at 95°C. Beads were separated from the complex by centrifugation (6000g x 5 minutes), and the protein was resolved in a 4-12% SDS-PAGE gel, transferred on to PVDF membrane and incubated with specific antibodies.

### Drug treatments and FACS analysis

Hepa1-6 and Huh-7 cells were treated with nocodazole (1 μM) and hydroxyurea (2.5 mM) for 24 hours. The cells were then trypsined, fixed in ice cold 70% ethanol, washed with cold PBS and stained with propidium iodide (PI)/RNAse buffer. The cell cycle profiles were determined with a FACSCalibur flow cytometry system, and CellQuest Pro Software (BD Biosciences) was used for acquisition and analysis.

### Antibodies and immunoblotting

Whole cell lysates of different cell lines and liver tissues of mice were prepared by RIPA lysis buffer (#89900, Thermo Fisher), supplemented with protease inhibitors (#11836153001, Roche). For Western blot analysis, 50 to 100 μg protein was resolved on NuPAGE precast gels (# NP0322BOX, Invitrogen), transferred using XCell II Blot module (#090707-098, Invitrogen) onto Immobilon-FL membranes (#IPVH07850, Millipore), and probed with specific primary antibodies. The following antibodies were used: Scrib (#sc-11048 (K-21), Santa Cruz), β-actin (#A5441, Sigma), Lamin a/c (#ab108922, Abcam), Na^+^/K^+^ ATPase (#ab7671, Abcam), H-RAS (#sc-520, Santa Cruz), KRAS (#NBP-1-58261, Novus Biologicals), AKT (#4691S, Cell Signaling), pAKT (#4060S, Cell Signaling), PDK-1(#3062P, Cell Signaling), pPDK-1(#3061S, Cell Signaling), β-catenin (#9562S, Cell Signaling), ERK-1/2 (#4695S, Cell Signaling), pERK-1/2 (#T202/Y204, 4370S, Cell Signaling), YAP/TAZ (#D24E4, 8418S, Cell Signaling), Cyclin D1 (#ab134175, Abcam), Cyclin E1(#13A3; sc-56310, Santa Cruz), p53 (#FL-393; sc-6243, Santa Cruz), PTEN (#9188S, Cell Signaling), Rb (#sc-50, Santa Cruz), pRb (#8516S, Cell Signaling), AMPK (#2532S, Cell Signaling), pAMPK (#2531S, Cell Signaling), RAF-1 (#C-12, sc-133, Santa Cruz), pRAF-1 (#2696S, Cell Signaling), B-RAF (#C-19, sc-166, Santa Cruz), p-B-RAF (#S445; 2696S, Cell Signaling), MEK1/2 (#9122S, Cell Signaling), pMEK1/2 (#sc-7995, Ser218/Ser222, Santa Cruz), MST-1 (#3682P, Cell Signaling) SAV-1 (#13301P, Cell Signaling) MOB-1 (#13730P, Cell Signaling), pLATS1 (#9157S, S909, Cell Signaling), pYAP-1(#13619P, Ser397, Cell Signaling), pYAP-1 (#13008P, Ser127, Cell Signaling) and Flag (#F3165, Sigma). All the antibodies from Santa Cruz were used at 1:500 dilution, whereas antibodies from Cell Signaling Technology (CST) and Abcam were used at 1:1000 dilutions. β-actin antibody was used at 1:10,000 dilution. The following secondary antibodies (IRDye-conjugated) were used: donkey anti-mouse IRDye 800CW (#926-32212), donkey anti-goat IRDye 800CW (#926-32214), donkey anti-rabbit IRDye 800CW (#926-32213; all from Li-Cor Biosciences). Li-Cor Biosciences Odyssey Classic Imager was used to scan and capture the Western blot images.

### Statistical analysis

All the experiments were performed in triplicate and were repeated at least three times wherever applicable. The data were presented as mean ± SD. Unpaired student *t-test* was used to compare data among groups, and *p value*s less than 0.05 were considered statistically significant.
